# Differentiation of the Emerging Human Pathogens *Trichosporon asahii* and *Trichosporon asteroides* from Other Pathogenic Yeasts and Moulds by Using Species-Specific Monoclonal Antibodies

**DOI:** 10.1371/journal.pone.0084789

**Published:** 2014-01-02

**Authors:** Genna E. Davies, Christopher R. Thornton

**Affiliations:** Biosciences, University of Exeter, Exeter, Devon, United Kingdom; University of Minnesota, United States of America

## Abstract

The fungal genus *Trichosporon* contains emerging opportunistic pathogens of humans, and is the third most commonly isolated non-candidal yeast from humans. *Trichosporon asahii* and *T. asteroides* are the most important species causing disseminated disease in immunocompromised patients, while inhalation of *T. asahii* spores is the most important cause of summer-type hypersensitivity pneumonitis in healthy individuals. Trichosporonosis is misdiagnosed as candidiasis or cryptococcosis due to a lack of awareness and the ambiguity of diagnostic tests for these pathogens. In this study, hybridoma technology was used to produce two murine monoclonal antibodies (MAbs), CA7 and TH1, for detection and differentiation of *Trichosporon* from other human pathogenic yeasts and moulds. The MAbs react with extracellular antigens from *T. asahii* and *T. asteroides*, but do not recognise other related *Trichosporon* spp., or unrelated pathogenic yeasts and moulds including *Candida, Cryptococcus*, *Aspergillus*, *Fusarium*, and *Scedosporium* spp., or the etiologic agents of mucormycosis. Immunofluorescence and Western blotting studies show that MAb CA7, an immunoglobulin G1 (IgG1), binds to a major 60 kDa glycoprotein antigen produced on the surface of hyphae, while TH1, an immunoglobulin M (IgM), binds to an antigen produced on the surface of conidia. The MAbs were used in combination with a standard mycological growth medium (Sabouraud Dextrose Agar) to develop an enzyme-linked immunosorbent assay (ELISA) for differentiation of *T. asahii* from *Candida albicans* and *Cryptococcus neoformans* in single and mixed species cultures. The MAbs represent a major advance in the identification of *T. asahii* and *T. asteroides* using standard mycological identification methods.

## Introduction

The genus *Trichosporon* contains approximately 50 species of basidiomycete yeasts found in a wide variety of habitats including soil and indoor environments [1]–[3]. A number of species colonise the skin, gastrointestinal, respiratory and urinary tracts of humans [2], [3], and superficial *Trichosporon* infections of immunocompetent individuals such as white piedra infections of the hair shaft are well documented [2], [3]. As well as superficial infections, repeated inhalation of *Trichosporon* arthroconidia can cause summer-type hypersensitivity pneumonitis (SHP) [4], [5], an immunologically induced lung disease. It is the most common form of hypersensitivity pneumonitis (HP) in Japan [4] and *T. asahii* is the most frequent cause of the disease [1], [3]. However, other fungi such as the basidiomycete yeast *Cryptococcus* may be responsible for SHP [6] and accurate identification of the causative species is necessary for appropriate treatment, which is challenging due to the number of different techniques required [7]–[10].

Certain *Trichosporon* species have also emerged as rare but frequently fatal pathogens causing disseminated infections (trichosporonosis) in immunocompromised individuals and ICU patients [2], [3], [11]–[18]. The genus *Trichosporon* was the third most commonly isolated non-candidal yeast from clinical specimens in the ARTEMIS DISK global antifungal surveillance study [14] and is the second most common cause, after *Candida* species, of yeast fungaemia in patients with haematological malignancies [2], [3]. High mortality rates are associated with trichosporonosis, with reports in the literature of between 50 and 80% in high-risk patient groups [2].

Early diagnosis of invasive trichosporonosis is critical for prompt and effective treatment [2], [3] but this is difficult for *Trichosporon* infections [17], especially in resource-limited settings with rudimentary diagnostic facilities. Currently, for a proven diagnosis of invasive trichosporonosis, the fungus must be cultured from biopsies [3], [17], but obtaining biopsy samples from critically ill patients is problematic. Furthermore, *Trichosporon asahii*, the most common cause of disseminated disease, may be mistaken for *Candida* spp. in culture, especially where mixed yeast or mould species are recovered [7], [19], [20], and methods for identifying *Trichosporon* to species level, based on morphological characteristics and biochemical profiling are time consuming, require specialist training, appropriately equipped laboratory facilities, and can yield inconsistent results [7]. Nucleic acid-based methods are necessary to distinguish *Trichosporon* spp., but no such methods have been standardised for clinical use and no commercially available tests currently accommodate the revised taxonomic classification of the genus [8]–[10], [21]. Cross-reactivity of commercial immunoassays with *Trichosporon,* such as the *Cryptococcus* antigen test [22], can also lead to mis-identification and inappropriate use of antifungal drugs.

Accurate and relatively simple diagnostic procedures are needed for *Trichosporon* detection [2], [3], [17]. Hybridoma technology allows the generation of highly specific monoclonal antibodies (MAbs) [23]–[25] that can be used to develop rapid and highly accurate immunodiagnostic tests capable of differentiating fungal pathogens to genus-, species- or even isolate-level [23]–[25]. This paper describes the use of hybridoma technology to develop two murine hybridoma cell lines (CA7 and TH1) producing MAbs specific for *Trichosporon asahii* and the closely related species *Trichosporon asteroides*, the most important causes of trichosporonosis. The accuracy of the MAbs in differentiating *T. asahii* from other yeast pathogens in mixed cultures is demonstrated using a highly specific ELISA combined with a standard mycological culture method.

## Materials and Methods

### Ethics Statement

All animal work described in this study was conducted under a UK Home Office Project License, and was reviewed by the institution’s Animal Welfare Ethical Review Board (AWERB) for approval. The work was carried out in accordance with The Animals (Scientific Procedures) Act 1986 Directive 2010/63/EU, and followed all the Codes of Practice which reinforce this law, including all elements of housing, care, and euthanasia of the animals.

### Fungal Strains and Culture Conditions

*Trichosporon asahii* is currently the most common cause of invasive trichosporonosis [2], [3], [15], [16]. Consequently, a clinical isolate of the fungus (*Trichosporon asahii* var. *asahii* CBS 8972)(Table 1) was used to prepare immunogen for immunisation of mice. The fungus was grown in malt yeast broth (MYB; bactopeptone 5 g l^−1^, malt extract 10 g l^−1^, glucose 40 g l^−1^, yeast extract 4 g l^−1^, pH to 7.3 with 1 M NaOH) or on malt yeast agar (MYA; MYB (Difco), agar 20 g l^−1^). Sabouraud dextrose agar (SDA; SD broth (Difco), agar 20 g l^−1^) was used in mixed species specificity screening tests. *Candida, Rhodotorula, Magnusiomyces, Kluyveromyces, Geotrichum, Wickerhamomyces* and *Pichia* species were all grown on Glucose-Peptone-Yeast extract agar (GPYA; glucose 40 g l^−1^, bacteriological peptone 5 g l^−1^, yeast extract 5 g l^−1^, agar 15 g l^−1^). *Aspergillus, Penicillium, Scedosporium, Paecilomyces,* and *Pseudallescheria* species were all grown on Malt Extract Agar (MEA; malt extract (Oxoid) 20 g l^−1^, agar 20 g l^−1^). *Malassezia furfur* was grown on Leeming-Notman agar (LNA; bacteriological peptone 10 g l^−1^, glucose 5 g l^−1^, yeast extract 0.1 g l^−1^, ox bile 8 g l^−1^, 0.0001% glycerol, glycerol *m*-monostearate 0.5 g l^−1^, 0.00005% Tween-60, 1.0% cow’s milk, agar 12 g l^−1^). All other fungi were grown on Potato Dextrose Agar (PDA; PDB (Sigma), agar 20 g l^−1^). All media were autoclaved at 121°C for 15 min before use and cultures grown at 26°C with a 16 h photoperiod of fluorescent light, with the exception of *M. furfur* which was grown at 30°C in the dark.

**Table 1:** Details of fungal cultures.

Organism	Isolate number	Source^a^
*Trichosporon asahii* var. *asahii*	8972	CBS
*Trichosporon asahii* var. *asahii*	8973	CBS
*Trichosporon asahii* var. *asahii*	5286	CBS
*Trichosporon asahii* var. *asahii*	7632	CBS
*Trichosporon asahii* var. *asahii*	5599	CBS
*Trichosporon asahii*	2479	CBS
*Trichosporon asteroids*	6183	CBS
*Trichosporon asteroides*	7623	CBS
*Trichosporon asteroides*	2481	CBS
*Trichosporon asteroides*	7624	CBS
*Trichosporon cutaneum*	2466	CBS
*Trichosporon dermatitis*	2043	CBS
*Trichosporon inkin*	7630	CBS
*Trichosporon inkin*	7655	CBS
*Trichosporon loubieri*	7065	CBS
*Trichosporon mucoides*	7625	CBS
*Trichosporon mycotoxinivorans*	9756	CBS
*Trichosporon ovoides*	7556	CBS
*Alternaria infectoria*	137.9	CBS
*Aspergillus cervinus*	537.65	CBS
*Aspergillus fumigatus*	AF293	SK
*Aspergillus nidulans*	A4	FGSC
*Aspergillus niger*	102.4	CBS
*Aspergillus oryzae*	AO1	CRT
*Aspergillus terreus* var. *terreus*	601.65	CBS
*Botrytis cinerea*	R2	CRT
*Candida albicans*	5314	SB
*Candida dubliniensis* var. *dubliniensis*	8500	CBS
*Candida glabrata*	4962	CBS
*Candida krusei*	5590	CBS
*Candida parapsilosis* var. *parapsilosis*	8836	CBS
*Candida tropicalis* var. *tropicalis*	1920	CBS
*Cryptococcus neoformans* (serotype D)	5728	CBS
*Cryptococcus neoformans* var. *neoformans*	7779	CBS
*Cryptococcus saitoi*	1975	CBS
*Cunninghamella elegans*	151.8	CBS
*Filobasidiella bacillispora*	10865	CBS
*Filobasidiella neoformans*	10490	CBS
*Filobasidiella neoformans*	10496	CBS
*Fusarium oxysporum* f.sp. lycopersici	167.3	CBS
*Fusarium solani*	224.34	CBS
*Geotrichum candidum*	115.23	CBS
*Kluyveromyces marxianus*	3073	CBS
*Lichtheimia corymbifera*	TJAFJ713070	CRT
*Magnusiomyces capitatus*	207.83	CBS
*Malassezia furfur*	9596	CBS
*Paecilomyces variotii* 10.1	10.1	CRT
*Penicillium islandicum*	338.48	CBS
*Pichia norvegensis*	6564	CBS
*Pseudallescheria boydii*	835.96	CBS
*Pythium ultimum* var. *ultimum*	656.68	CBS
*Rhizomucor miehei*	360.92	CBS
*Rhizopus stolonifer* var. *stolonifer*	389.95	CBS
*Rhodosporidium toruloides*	6016	CBS
*Rhodotorula mucilaginosa* var. *mucilaginosa*	326	CBS
*Scedosporium apiospermum*	117407	CBS
*Scedosporium prolificans*	467.74	CBS
*Sporidiobolus salmonicolor*	6781	CBS
*Verticillium dahliae*	178.66	CBS
*Wickerhamomyces anomalus*	5759	CBS

^a^ CBS; Centraalbureau voor Schimmelcultures, Utrecht, The Netherlands. SK; S. Krappman, Institute of Microbiology and Genetics, Department of Molecular Microbiology and Genetics, Georg-August University, Gottingen, Germany. FGSC; Fungal Genetics Stock Centre, University of Missouri, Kansas City. CRT; C.R.Thornton, University of Exeter, UK. SB; S.Bates, University of Exeter, UK.

### Preparation of Immunogen, Immunisation Regime, and Animal Welfare

For preparation of the immunogen, flasks containing 100 ml of sterile MYB were inoculated with 10^3^ spores of *T. asahii* ml^−1^ of medium. The flasks were incubated at 26°C with shaking (125 rpm) for 2 d, after which the contents were centrifuged at 4000 rpm for 5 min, the bulk of the supernatant was discarded and the pelleted cells re-suspended in 10 ml of remaining culture fluid. The surface of 2-day-old MYA petri-dish cultures were scraped and combined with the re-suspended cells. The combined cell preparations were snap frozen in liquid nitrogen, lyophilized for 3 d and the dried material stored at −20°C prior to use. Before immunisation, the immunogen was reconstituted with phosphate buffer saline (PBS; 137 mM NaCl, 2.7 mM KCl, 8 mM Na_2_HPO_4_, and 1.5 mM KH_2_PO_4_ [pH 7.2]) to make a cell suspension containing 10 mg biomass ml^−1^ buffer. For immunisations, 6-week-old BALB/c white mice were each given four intraperitoneal injections (300 µl per injection) of immunogen at 2-week intervals and a single booster injection was given five days before fusion.

### Production and Screening of Hybridomas and Determination of Antibody Specificities

Hybridoma cells were produced by the method described elsewhere [24], [25] and the supernatants were screened by enzyme-linked immunosorbent assay (ELISA) against antigens immobilized to the wells of Maxisorp microtitre plates (442404; Nunc) (50 µl per well). For antibody specificity tests, antibodies were tested against surface washings [25] prepared from replicate slant cultures of fungi. Protein concentrations, determined spectrophotometrically at 280 nm (Nanodrop, Agilent Technologies Limited, Berkshire, UK), were adjusted with PBS to produce equivalent protein concentrations for each organism. Fifty µl volumes were then used to coat the wells of microtitre plates. After incubating overnight at 4°C, wells were washed four times with PBST (PBS containing 0.05% Tween-20) and once each with PBS and dH_2_O and air-dried at 23°C in a laminar flow hood. The plates were stored in sealed plastic bags at 4°C in preparation for screening of hybridoma supernatants by ELISA.

### Enzyme-Linked Immunosorbent Assay

Wells containing immobilised antigens were blocked for 15 min with 100 µl of PBS containing 1% Bovine Serum Albumin (BSA) and, after one 5-min wash with PBS, were incubated successively with hybridoma supernatant for 1 h, followed with goat anti-mouse polyvalent (immunoglobulin classes IgG, IgA, and IgM) peroxidase conjugate (A-0412; Sigma Chemical Company, Poole, United Kingdom) diluted 1 in 1000 in PBST for a further hour. Bound antibody was visualized by incubating wells with tetramethyl benzidine (T-2885; Sigma) substrate solution [24], [25] for 30 min. The reactions were stopped by the addition of 3 M H_2_SO_4_. Absorbance values were determined at 450 nm with an automated microplate reader (iMark™ microplate reader MPM6, BIORAD, Hertfordshire, UK). Wells were given four 5-min rinses with PBST between incubations. Working volumes were 50 µl per well, and control wells were incubated with tissue culture medium (TCM) containing 10% fetal bovine serum. All incubation steps were performed at 23°C in sealed plastic bags. The threshold for detection of the antigen in ELISA was determined from control means (2×TCM absorbance values) [25]. These values were consistently in the range 0.050–0.100. Consequently, absorbance values >0.100 were considered as positive for the detection of antigen.

### Determination of Ig Subclass and Cloning Procedure

The Ig class of MAbs was determined by using antigen-mediated ELISA. Wells of antigen-coated microtitre plates were incubated successively with hybridoma supernatant for 1 h, followed with goat anti-mouse IgG_1_, IgG_2a_, IgG_2b_, IgG_3_, IgM, or IgA-specific antiserum (ISO-2; Sigma) diluted 1 in 3000 in PBST for 30 min and rabbit anti-goat peroxidase conjugate diluted 1 in 1000 (A-5420; Sigma) for a further 30 min. Bound antibody was visualized with TMB substrate as described. Hybridoma cells lines were sub-cloned three times by limiting dilution, and cell lines were grown in bulk in a non-selective medium preserved by slowly freezing in foetal bovine serum/dimethyl sulfoxide (92∶8), and stored in liquid nitrogen.

### Antigen Characterisation by Heat Treatment, Periodate Oxidation and Protease Digestion

Heat stability studies were conducted by placing tubes of solubilised antigen from three replicate cultures of the fungus in a boiling water bath. At 10 min intervals, samples were removed, centrifuged at 14,500 rpm for 5 min, and antigens immobilised to the wells of microtitre plates for assay by ELISA as described. For periodate oxidation, microtitre wells containing immobilised antigens were incubated with 50 µl of sodium *meta*-periodate solution (20 mM NaIO_4_ in 50 mM sodium acetate buffer [pH4.5]) or acetate buffer only (control) at 4°C in sealed plastic bags. Plates were given four 3-min PBS washes before processing by ELISA as described. For protease digestions, microtitre wells containing immobilised antigen were incubated with 50 µl of pronase (protease XIV; 9 mg ml^−1^ in PBS) or trypsin (1 mg ml^−1^ in Milli-Q H_2_O) solution or Milli-Q H_2_O or PBS only (controls) for 4 h at 37°C or 4°C. Plates were given four 3-min rinses with PBS and then assayed by ELISA as described.

Protease digestion studies were also conducted using DOT-BLOTS of antigen immobilised to polyvinylidene difluoride (PVDF) membranes. Membranes were incubated in pronase or trypsin solutions or controls for 4 h at 37°C with shaking (25 rpm). Membranes were washed three times with PBS and then blocked for 16 h at 4°C, with PBS containing 1% BSA. Blocked membranes were incubated with MAb supernatant diluted 1 in 2 with PBS containing 0.5% BSA (PBSA), for 2 h at 23°C. After washing three times with PBS, membranes were incubated for 1 h with goat anti-mouse alkaline phosphatase conjugate, diluted 1 in 15,000 in PBSA (either IgM μ-chain specific, Sigma; A9688 or IgG whole molecule, Sigma; A3562). Membranes were washed three times with PBS, once with PBST and bound antibody visualised by incubation in substrate solution [24], [25]. Reactions were stopped by immersing membranes in dH_2_O, and membranes were then air dried between sheets of Whatman filter paper.

### Polyacrylamide Gel Electrophoresis and Western Blotting

SDS-PAGE was carried out using 4–20% gradient polyacrylamide gels under denaturing conditions. Surface washings of *T. asahii* var. *asahii* CBS8972, *T. asteroides* CBS6183 and *T. inkin* CBS7630 cultures grown on MYA slants were prepared every 24 h following inoculation, using 3 ml of sterile Milli-Q water. Washings were centrifuged for 5 min at 14,500 rpm to precipitate cells and hyphae and the supernatants containing soluble antigens were denatured by mixing with Laemmli buffer and heating at 95°C for 10 min. Proteins were separated electrophoretically at 165 V and pre-stained, broad-range markers (Bio-Rad Laboratories Limited, Hemel Hempstead, UK) were used for molecular weight determinations. For Western blotting, separated proteins were transferred electrophoretically on to a PVDF membrane for 2 h at 75 V. Thereafter, the membranes were processed with MAb supernatants according to the procedure used for DOT-BLOTS.

### Immunofluorescence

Sterilised slides were coated with a washed yeast cell suspension containing 1% glucose and incubated at 26°C for 16 h. After air-drying, the slides were fixed and incubated with hybridoma supernatant for 1 h, followed by three 5 min PBS washes. Slides were then incubated with goat anti-mouse polyvalent fluorescein isothiocyanate conjugate (diluted 1 in 40 in PBS) (Sigma; F1010) for 30 min. Slides were given three 5 min washes with PBS and mounted in PBS-glycerol mounting medium before overlaying with coverslips. All incubation steps were performed at 23°C in a humid environment to prevent evaporation and slides were stored in the dark, at 4°C, prior to examination using an epifluorescence microscope (Olympus IX81).

### Differentiation of *Trichosporon* from *Candida* and *Cryptococcus* in Mixed Species Cultures

Petri dish culture plates containing SDA were inoculated with cell suspensions of *T. asahii*, *C. albicans* or *C. neoformans*, either as single species cultures or as species mixtures. After 24 h incubation at 26°C, antigen solutions were prepared by flooding the plates with 10 ml PBS, suspending cells using sterile L-shaped spreaders, and pelleting of cells by centrifugation at 14,500 rpm for 5 min. Protein concentrations of solutions were adjusted to 60 µg ml^−1^ and used to coat the wells of microtitre plates for assay by ELISA.

### Data Analysis

Differences in means were analysed by one-way analysis of variance (ANOVA) and Tukey-Kramer tests were used to determine statistical significance.

## Results

### Production of Hybridoma Cell Lines and Isotyping of MAbs

Two fusions were performed and 1284 hybridoma cell lines were screened for MAb production by PTA-ELISA. Two of the MAbs (CA7 and TH1) were selected for further testing on the basis of their high absorbance values (A_450_ 0.400 and A_450_ 1.122 respectively). Isotyping of the MAbs showed that CA7 belongs to immunoglobulin class G1 (IgG1) and TH1 to immunoglobulin class M (IgM).

### MAb Specificity Tests

**Figure 1:**
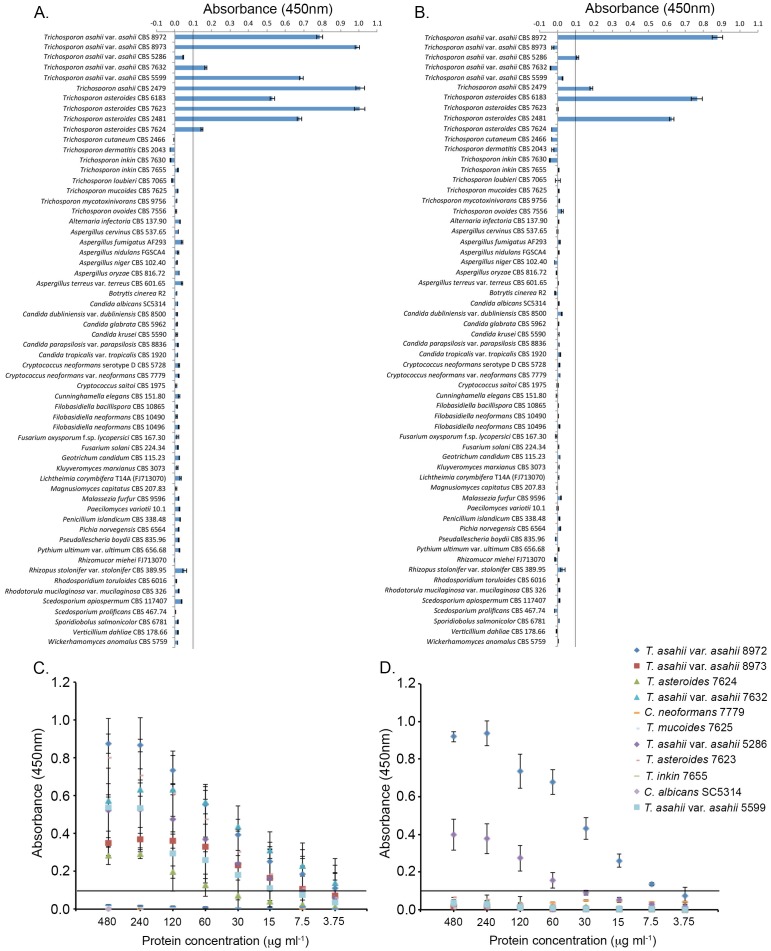
ELISA absorbance values from specificity screening tests using MAbs CA7 (A and C) and TH1 (B and D), using an antigen protein concentration of 60 µg ml^−1^ (A and B) and a range of protein concentrations from 3.75 µg ml^−1^ to 480 µg ml^−1^ (C and D) for each of the organisms tested. Bars (Figs. A and B) and symbols (C and D) are the means of four and three biological replicates respectively ± standard errors. The threshold absorbance value for detection of antigen in ELISAs is ≥0.100 (indicated by lines on graphs).

In ELISA specificity tests, using antigen protein concentrations of 60 µg ml^−1^, MAbs CA7 and TH1 reacted against surface antigens from *T. asahii* and *T. asteroides*, but did not react with surface antigens from other *Trichosporon* species or a broad range of clinically relevant yeasts and moulds including *Candida, Cryptococcus* and *Aspergillus* species (Table 1). While MAb CA7 failed to recognise *T. asahii* var. *asahii* CBS 5286 at this protein concentration, MAb TH1 gave a positive absorbance value (Abs_450_>0.100) for this strain. Consequently, when used together at this protein concentration, the MAbs gave a combined positive reaction with all of the *T. asahii* and *T. asteroides* strains tested (Figs. 1A and 1B). When tested against higher concentrations of protein (>60 µg ml^−1^), both MAbs reacted strongly with *T. asahii* CBS 5286 (Figs. 1C and 1D), while concomitant detection of other *Trichosporon* species (*T. mucoides* and *T. inkin*), and the unrelated pathogens *Candida* and *Cryptococcus* did not occur at equivalent concentrations of protein (Figs. 1C and 1D). Despite this, TH1 detection of *T. asahii* 8973, *T. asahii* 7632, *T. asahii* 5599, and *T. asteroides* strains 7623 and 7624 did not occur at the higher concentrations of protein, while MAb CA7 detected all five of these strains at protein concentrations ≥60 µg ml^−1^. Consequently, the use of both antibodies at protein concentrations in the range 60 µg ml^−1^ to 480 µg ml^−1^ would allow for unequivocal detection of *T. asahii* and *T. asteroides* and differentiation from other infectious fungi.

### Heat, Chemical and Enzymatic Characterisation of Antigens

**Figure 2:**
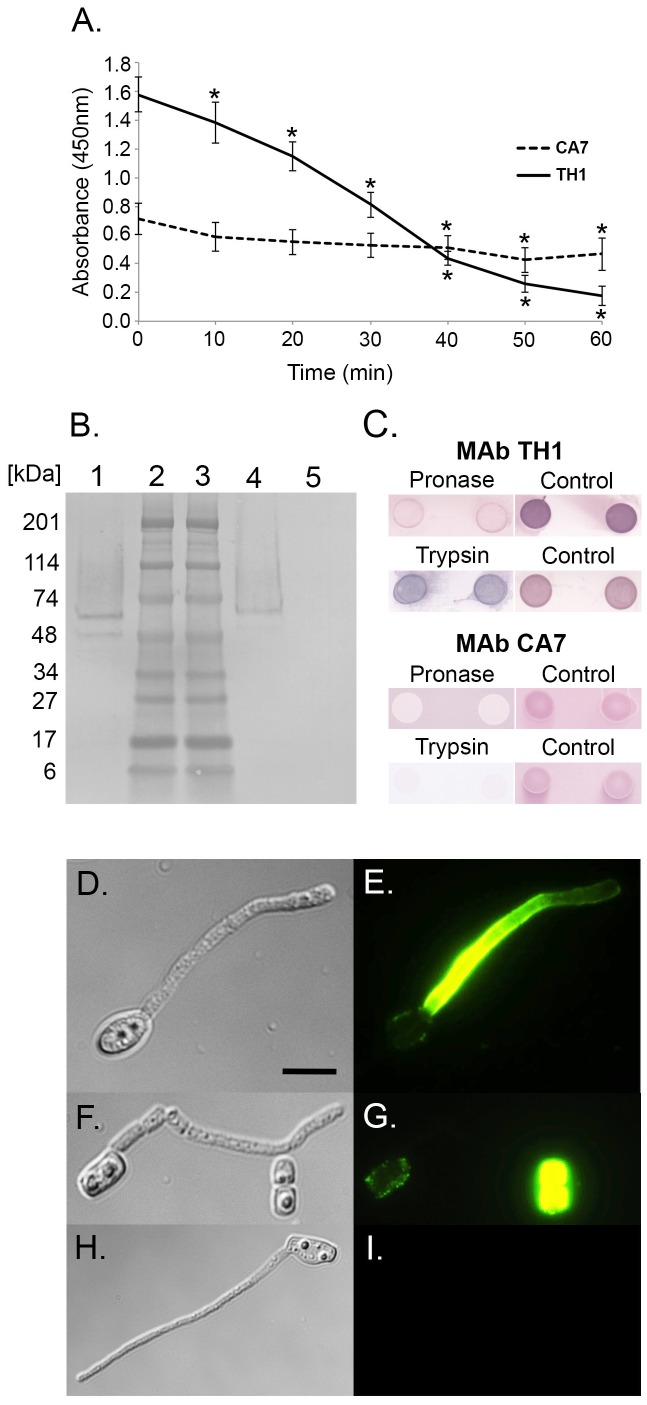
Characterisation of antigens bound by MAbs CA7 and TH1. (A) ELISA of heat-treated antigens using MAb CA7 (broken line) and MAb TH1 (continuous line). Each point is the mean of three biological replicates ± standard errors. Asterisks show significant (p<0.001) decrease in absorbance value compared to respective controls (time point zero). (B) Western immunoblot with MAb CA7 using antigens from 3-day-old cultures of *T. asteroides* (lane 1), *T. asahii* (lane 4) and *T. inkin* (lane 5). Wells were loaded with 1.6 µg of protein. Lanes 2 and 3 contain broad range molecular mass markers (kDa); (C) DOT-BLOTS of *T. asahii* antigen treated with pronase or trypsin and then processed with MAbs TH1 or CA7. Each DOT-BLOT consists of antigen from two biological replicates. (D–I) Photomicrographs of *T. asahii* var. *asahii* CBS8972 cells, immunostained with MAb CA7 (D, E), TH1 (F, G) or TCM only (H, I) and anti-mouse polyvalent Ig fluorescein isothiocyanate. (D) Bright field image of germinated conidium with hypha; (E) Same field of view as panel D but examined under epifluorescence. Note the intense staining of the hyphal cell wall but not the conidiium. (F) Bright field image of germinated conidium with hypha and ungerminated conidia. (G) Same field of view as panel F but examined under epifluorescence. Note intense staining of ungerminated conidial cell wall but not hypha, and reduced fluorescence of germinated conidium. (H) Bright field image of germinated conidium with hypha. (I) Same field of view as panel H but examined under epifluorescence. Bar, 8 µm.

The *T. asahii* var. *asahii* CBS8972 antigens were subjected to different treatments including heat (Fig. 2A) and chemical and enzymatic modifications (Tables 2 & 3). Reductions in MAb binding in ELISA following heating show that an epitope is heat labile. Reductions in binding following treatment with pronase show that the epitope consists of protein, while reductions with trypsin show a protein epitope contains positively charged lysine and arginine side chains. Reductions in antibody binding following chemical digestion of an antigen with periodate shows that its epitope is carbohydrate and consists of vicinal hydroxyl groups.

**Table 2:** Absorbance values from ELISA tests with MAbs CA7 and TH1 using periodate-treated antigens.

MAb	Time (h)	Absorbance (450 nm)	
		Periodate	Control
CA7	0	0.787±0.014	0.799±0.017
	1	0.757±0.018	0.769±0.018
	2	0.764±0.021	0.755±0.012
	3	0.730±0.011	0.756±0.010
	4	*0.696±0.009	0.793±0.023
	23	*0.601±0.009	0.734±0.012
TH1	0	1.196±0.018	1.247±0.029
	1	1.151±0.026	1.217±0.024
	2	1.156±0.017	1.210±0.027
	3	1.109±0.025	1.222±0.026
	4	1.084±0.017	1.179±0.032
	23	*0.861±0.030	1.144±0.018

Absorbance value significantly different (p<0.001) to control using ANOVA. Each value is the mean of eight biological replicates ± standard error.

**Table 3:** Absorbance values from ELISA tests with MAbs CA7 and TH1 using protease-treated antigens.

MAb	Temp(°C)	Absorbance (450 nm)			
		Pronase	Control	Pronase	Control
CA7	4	*0.281±0.015	0.612±0.025	0.427±0.017	0.468±0.019
	37	*0.323±0.020	0.592±0.029	*0.295±0.025	0.524±0.017
TH1	4	1.068±0.021	1.128±0.024	1.079±0.026	1.135±0.044
	37	*1.052±0.021	1.261±0.020	1.135±0.030	1.103±0.036

Absorbance value significantly different (p<0.001) to controls (buffer only) using ANOVA. Each value is the mean of eight biological replicates ± standard error.

The epitope bound by MAb CA7 was heat stable, with significant (p<0.001) reductions in ELISA absorbance only occurring after 40 min of heating (Fig. 2A). CA7 binding to its epitope was also significantly reduced (p<0.001) following periodate treatment for 4 and 23 h (Table 2), pronase treatment at 37°C and 4°C, and trypsin treatment at 37°C (Table 3) when compared to controls. Binding of TH1 to its target antigen was significantly (p<0.001) reduced following heat treatment for 10 min (Fig. 2A), periodate treatment for 23 h (Table 2) and pronase treatment at 37°C (Table 3). Trypsin treatment of the antigen did not significantly reduce TH1 binding (Table 3). DOT-BLOT studies of protease treated antigen showed similar results to ELISA tests. TH1 reactivity with antigen immobilised to PVDF membrane was reduced following treatment with pronase, but was unaffected by trypsin. CA7 reactivity was reduced by both proteases. The relative insensitivities of the MAbs to periodate treatment, but sensitivities to protease digestion in both ELISA and DOT-BLOT tests indicate that MAbs CA7 and TH1 bind to protein epitopes in heat stable and heat labile glycoprotein antigens respectively.

### Western Blotting and Immunofluorescence

Monoclonal antibody CA7 reacted with immuno-reactive glycoprotein antigen(s) from *T. asahii* and *T. asteroides*, with molecular masses in the region 48 to 74 kDa, and with a major glycoprotein antigen of approximately 60 kDa (Fig. 2B). These immuno-reactive antigens were absent in *T. inkin* antigen extracts. Monoclonal antibody TH1 did not bind to immuno-reactive antigen(s) in Western blotting studies. Immunofluorescence studies showed that MAb CA7 binding was specific to the surface of hyphae (Fig. 2D), whereas TH1 binding was specific to the surface of conidia (Fig. 2F).

### Differentiation of *Trichosporon* from *Candida* and *Cryptococcus* in Mixed Species Cultures

**Figure 3:**
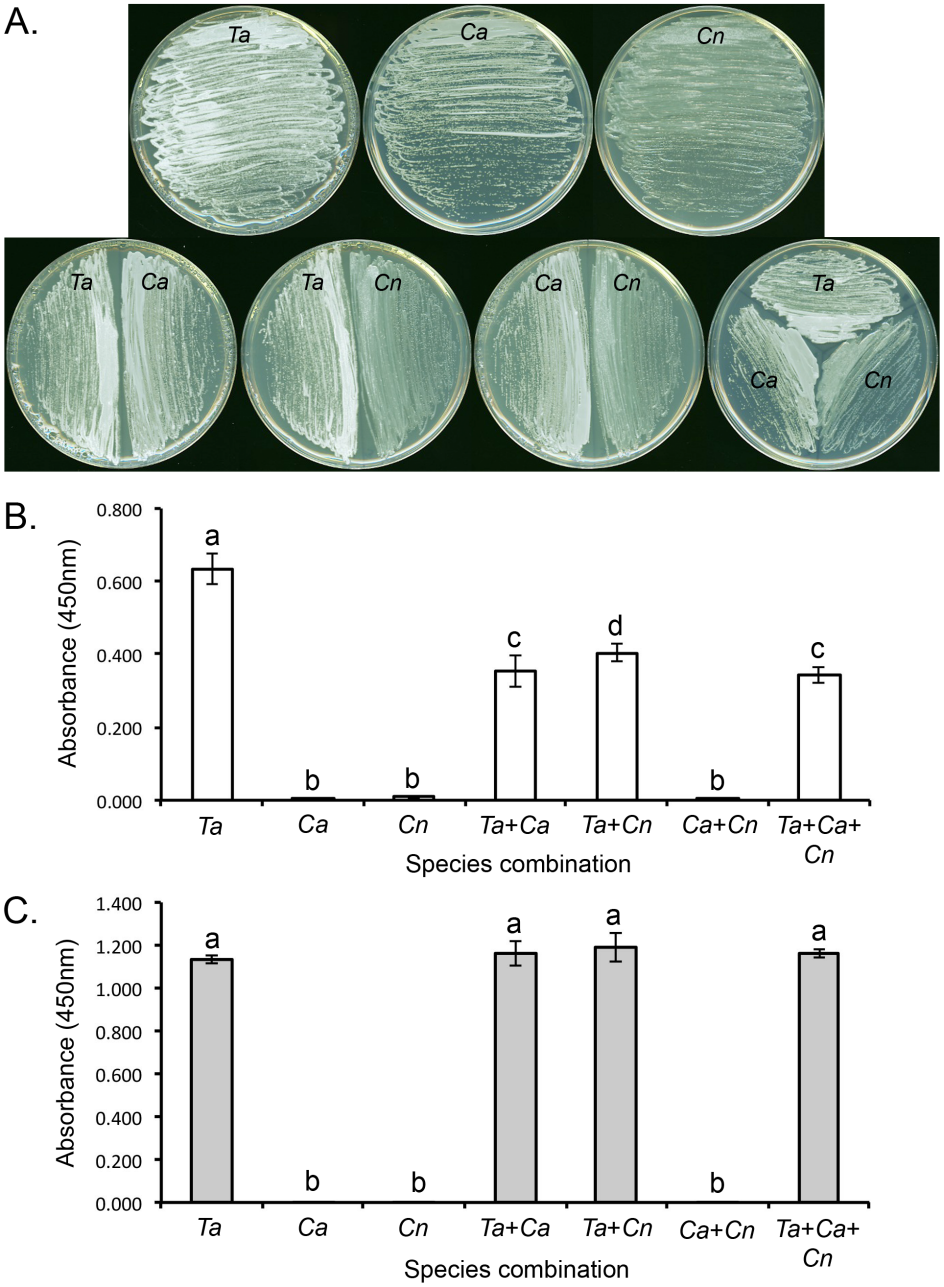
Growth of yeasts for 24 h as single species or mixed species cultures and ELISA tests of soluble antigens using MAbs CA7 and TH1. (A) Sabouraud Dextrose Agar plates inoculated with different combinations of species: *T. asahii* var. *asahii* CBS8972 (*Ta*), *C. albicans* SC5314 (*Ca*), and *C. neoformans* var. *neoformans* CBS7779 (*Cn*). ELISA absorbance values of antigen solutions tested using MAb CA7 (B) and MAb TH1 (C). Each bar is the mean of three biological replicates ± standard error. Bars with the same letter are not significantly different at p<0.001 (ANOVA and Tukey-Kramer test).

Yeasts were cultured on SDA plates for 24 h (Fig. 3A) and antigen solutions tested by ELISA using both MAbs (Fig. 3B&C). Monoclonal antibodies CA7 (Fig. 3B) and TH1 (Fig. 3C) were highly accurate in detecting *T. asahii* both when grown in single culture, and when grown on plates containing mixed populations of *C. albicans* and *C. neoformans.* The MAbs did not cross react with *C. albicans* or *C. neoformans*, when grown either as single species or as mixed populations.

## Discussion

This paper describes the production of two murine MAbs, CA7 and TH1, raised against surface antigens from *Trichosporon asahii*. Specificity tests showed that both MAbs reacted only with antigens from the species *Trichosporon asahii* and *Trichosporon asteroides.* When combined in an ELISA, the MAbs were able to detect all of the isolates of *T. asahii* and *T. asteroides* tested. The MAbs did not cross-react with other clinically important yeasts and moulds, demonstrating their exquisite specificity.

*Trichosporon asahii* is the principal etiologic agent of trichosporonosis in humans [2], [3]. While *T. asteroides* was originally described as a pathogenic agent of superficial infections [26], its prevalence as an agent of disseminated infection is increasing, since the first recorded case of *T. asteroides* trichosporonosis in 2002 [26]. Currently, the species is reported to be second only to *T. asahii* as the cause of trichosporonosis in immunocompromised patients [2], [3]. Consequently, MAbs CA7 and TH1 represent useful diagnostic reagents for the immunological detection of the most important agents of this disease.

Recognition of these two species, but lack of recognition of the other *Trichosporon* species tested, may be explained by their close phylogenetic relatedness. Phylogenetic studies of the *Trichosporon* genus have shown that *Trichosporon asahii* and *Trichosporon asteroides* belong to a distinct clade, clade ovoides [21]. Comparisons of their ITS (internal transcribed spacer) regions show 98.7% similarity and for the intergenic spacer 1 (IGS1) region, 75.1% similarity [21]. When the mitochondrial cytochrome *b* genes of the species are compared, DNA sequences are synonymous and differ by only 4.0%, meaning that the two species contain proteins with identical amino acid sequences [27].

Incorrect diagnosis of invasive trichosporonosis as candidiasis or cryptococcosis is a critical factor in the successful treatment of infected patients [3], since these fungi differ in their susceptibilities to the different classes of antifungal drugs used to treat human mycoses [3], [14], [17], [18], [21], [28], [29]. Cross-reactivity of the cryptococcal latex agglutination test with *Trichosporon* species can lead to incorrect identification and inappropriate treatment [22]. *Trichosporon* and *Cryptococcus* both secrete glucuronoxylomannans (GXM) and common domains are shared between the polysaccharides of the two species, forming antigens detected by the test [22]. Cryptococcal meningitis is recognised as an AIDS defining illness and the most common cause of adult meningitis [30]. Although *Cryptococcus neoformans* is the species most frequently detected in meningitis patients, other yeasts are isolated, including emergent human pathogens such as *Trichosporon* and *Rhodotorula* [2], [30]. Unlike the MAb used in the cryptococcal diagnostic test, MAbs CA7 and TH1 did not cross-react with other yeast species including *C. neoformans* and *Rhodotorula*, and so eliminate any ambiguity in species identification.

Western blotting studies showed that CA7 binds to a major glycoprotein antigen with a molecular weight of approximately 60 KDa, while immunofluorescence tests showed that the MAbs recognise surface antigens present on different morphological structures of *Trichosporon asahii*. Binding of MAb CA7 was specific to antigen(s) present on the surface of hyphae, whereas TH1 binding was specific to the surface of conidia. When combined, the MAbs are able to recognise both yeast and filamentous forms of the fungus, an important characteristic given that both of these stages of the pathogen are implicated in tissue invasion in disseminated infections [2], [3]. The different morphologies have also been seen in the formation of plaques [18] which are an important risk factor associated with invasive trichosporonosis and, when present on devices such as catheters, allow the fungus to evade treatment with yeast active drugs.

The increasing prevalence of patients with mixed yeast infections, particularly polymicrobial ones [7], [19], [20], [30], and problems of identifying the causative agents in mixed species cultures motivated us to test the accuracy of the MAbs in detecting *Trichosporon* in mixed populations with *Candida* and *Cryptococcus*. Diagnostic laboratories often favour the use of selective media such as CHROMagar Candida, designed to aid the identification and differentiation of yeasts by the presence of chomogenic substrates in the media that react with species-specific enzymes, producing different coloured colonies [7]. However, limitations of the CHROMagar medium exist, for example it struggles to detect mixed yeast cultures if one species is significantly more prevalent than others showing only the colour of the dominant phenotype [7]. Consequently, it is recommended that the medium is combined with other culture media for isolation of species [7], and remains a presumptive test requiring further mycological analysis for definitive species identification [7]. To improve the diagnostic accuracy of plate cultures, we used the standard medical mycology growth medium SDA to produce mixed cultures of *Trichosporon*, *Candida* and *Cryptococcus*, from which simple antigen solutions could be prepared for testing in an ELISA with MAbs CA7 and TH1. The ELISA results show that both MAbs correctly recognised the presence of *T. asahii* when grown individually or in combination with *C. albicans* and *C. neoformans,* thereby dramatically improving the diagnostic accuracy of the *in* *vitro* culture procedure.

It is not known at present whether the MAbs can be used to detect *T. asahii* and *T. asteroides* infections without the need for culture of the organisms *in* *vitro*. If the diagnostic antigens are present as circulating markers in the bloodstream during disseminated infections or, in the case of SHP, bronchoalveolar lavage fluids (BALf), then the potential exists for the development of point-of-care tests such as lateral-flow devices (LFDs) that can be used to rapidly detect fungal infections without the need for sophisticated laboratory facilities [23], [24]. Use of BALf has been demonstrated in the diagnosis of SHP, through the detection of fungal DNA [8], and a similar method might also be used for the immunodetection of *T. asahii* diagnostic antigens in SHP patients. We have already demonstrated here that the antibodies can be used in a ELISA procedure to improve the diagnostic accuracy of a simple culture procedure employing a standardised growing medium, but the immunoassay test time could be reduced further, from hours for the ELISA to minutes for an LFD [23], [24].

It is important to note that while the MAbs developed here detect the two most common causes of invasive trichosporonosis (*T. asahii* and *T. asteroides*) and the most common agent of SHP (*T. asahii*), the other species implicated in superficial and invasive infections (a continually expanding list including *T. inkin* and *T. cutaneum* [2], [3]) and SHP (currently the only other member of the genus noted as a causative agent is *T. mucoides* [3], [5]) would require differentiation with PCR sequencing of DNA sequences that have sufficient species differences such as the IGS1 region [21]. Nevertheless, the culture-ELISA technique described here represents a significant improvement in the accurate detection and differentiation of *Trichosporon* from other clinically important yeast pathogens such as *Candida* and *Cryptococcus*.
